# Prolonged Detection of Bovine Viral Diarrhoea Virus Infection in the Semen of Bulls

**DOI:** 10.3390/v12060674

**Published:** 2020-06-22

**Authors:** Andrew J. Read, Sarah Gestier, Kate Parrish, Deborah S. Finlaison, Xingnian Gu, Tiffany W. O’Connor, Peter D. Kirkland

**Affiliations:** Virology Laboratory, Elizabeth Macarthur Agriculture Institute, Woodbridge Road, Menangle, New South, Wales 2568, Australia; andrew.j.read@dpi.nsw.gov.au (A.J.R.); sarah.gestier@dpi.nsw.gov.au (S.G.); kate.parrish@dpi.nsw.gov.au (K.P.); deborah.finlaison@dpi.nsw.gov.au (D.S.F.); xingnian.gu@dpi.nsw.gov.au (X.G.);

**Keywords:** bovine viral diarrhoea virus, Pestivirus, persistent testicular infection, prolonged testicular infection, bovine, testes, semen

## Abstract

Infection of bulls with bovine viral diarrhoea virus (BVDV) can result in the development of virus persistence, confined to the reproductive tract. These bulls develop a normal immune response with high neutralizing antibody titres. However, BVDV can be excreted in the semen for a prolonged period. Although relatively rare, in this study we describe six separate cases in bulls being prepared for admission to artificial breeding centres. Semen samples were tested in a pan-Pestivirus-reactive real-time PCR assay and viral RNA was detected in semen from five of the bulls for three to eight months after infection. In one bull, virus was detected at low levels for more than five years. This bull was found to have one small testis. When slaughtered, virus was only detected in the abnormal testis. The low levels of BVDV in the semen of these bulls were only intermittently detected by virus isolation in cell culture. This virus-contaminated semen presents a biosecurity risk and confirms the need to screen all batches of semen from bulls that have been previously infected with BVDV. The use of real-time PCR is recommended as the preferred laboratory assay for this purpose.

## 1. Introduction

Pestiviruses are a group of viruses that belong to the family Flaviviridae and are of animal health and economic importance worldwide [1]. The taxonomy of the Pestivirus genus has been reorganised to 11 species based on nucleotide and deduced amino acid sequence relatedness, antigenic similarity and host of origin [2,3] Bovine viral diarrhoea virus type 1 (BVDV-1) has been taxonomically classified as *Pestivirus A,* and BVDV type 2 (BVDV-2) has been classified as *Pestivirus B*. These viruses are predominantly pathogens of cattle, though sheep, other ruminants and pigs may also become infected.

Transmission of BVDV occurs horizontally by direct and indirect contact between cattle, and vertically by transplacental infection of the foetus. The virus replicates in mucosal epithelium and associated lymphoid tissues. A viraemia follows with subsequent viral replication throughout the body. Post-natal transient infections are often subclinical, but can result in a range of clinical conditions including reproductive losses, immune suppression, respiratory disease and acute diarrhoea [4]. Periconceptional infections can result in conception failure and embryonic deaths. Gestational infections can result in foetal death, congenital abnormalities, or a calf that becomes persistently infected (PI) for life [4].

The detection of BVDV in the semen of bulls is well documented [5,6,7,8,9,10]. Contamination of semen can arise from three types of infection. Firstly, male calves exposed to BVDV in utero, before they become immunocompetent, are born PI and will shed virus from most organs including in the semen for life [11,12]. These calves are born immunotolerant to BVDV and are often weak, ill-thrifty and are frequently infertile. However, occasionally PI bulls may grow normally, have semen of normal quality and can even gain entry to an artificial breeding centre [13]. There is usually a high concentration of BVDV in the semen of PI bulls (10^4^ to 10^7.6^ TCID_50_/mL) [5,9,14]. Semen with high titres of virus can infect susceptible cows following insemination [15,16].

Secondly, post-pubertal bulls exposed to BVDV undergo a transient infection and may shed virus in semen for up to 14 days [9], or perhaps 28 days [17] following infection. Virus levels tend to be low (10^0.9^ to 10^1.8^ TCID_50_/mL) but can occasionally infect a susceptible female [9]. These bulls will eventually mount an immune response to the virus which prevents further shedding.

Thirdly, some post-pubertal bulls exposed to BVDV become acutely infected but develop a prolonged or perhaps persistent testicular infection. These bulls are not PI, become transiently infected and mount a normal antibody response. The virus is cleared systemically, yet the virus is excreted in semen for prolonged periods. For this study, we have defined this prolonged testicular infection as the detectable presence of BVDV RNA in semen more than 60 days from when the systemic infection occurred. Until recently, these prolonged testicular infections (PTI) were considered to be rare. The first case was reported in 1998 [18]. This bull was not viraemic and therefore not PI. Nevertheless, infectious BVDV was detected in his semen at a moderate level (10^3.3^ TCID_50_/mL) for the rest of his life.

PTIs were induced experimentally in 2003 by inoculating susceptible post-pubertal bulls with BVDV [14]. Following acute infection, the bulls appeared to mount a typical immune response with the cessation of detectable viraemia. BVDV RNA was detected in the semen of these bulls by reverse transcription-nested PCR for a prolonged period of time. Attempts to isolate BVDV from the semen of these bulls were unsuccessful in cell culture, however semen collected five months after inoculation established infection in a naïve calf when given intravenously. These results were replicated by the same research team [6]. While the aetiology of PTIs with BVDV is still poorly understood, administration of a live attenuated vaccine virus has also been shown to induce PTI [7].

The incidence of PTI is considered to be very low and few have been described [17,18,19], yet due to the economic significance of BVDV infection, the World Organization for Animal Health (OIE) recommends that semen from seropositive bulls be screened for the presence of BVDV [20]. Before entry to a semen collection centre, bulls are tested to demonstrate that they are not viraemic or PI. Bulls that give negative results are then held in isolation at least 28 days [21] to undertake pre-entry testing and to ensure that animals recently exposed to BVDV can be reliably identified by allowing time for seroconversion. Once admitted to the semen collection centre, semen collections from all bulls with neutralising antibodies to BVDV are tested by a real-time, reverse transcription PCR (qRT-PCR). Following the introduction of this requirement, we have identified six bulls that have exhibited prolonged or persistent testicular infection. The detailed investigation and subsequent longitudinal sampling of these bulls is described.

## 2. Materials and Methods

### 2.1. Specimens

Blood samples and commercially prepared, extended semen from six bulls were submitted to the Virology Laboratory at the Elizabeth Macarthur Agriculture Institute (EMAI), New South Wales, Australia by veterinarians from artificial breeding centres. These samples were tested to meet regulatory protocols for the export of semen, where the serological status of an animal is determined and, if seropositive, their semen was subjected to virus detection. Subsequent to testing of the initial samples from these bulls, both archival samples and prospective collections were requested.

Bull 2 was sent to an abattoir where the testes were collected and sent to EMAI. The testes were measured, weighed and dissected. Cut surfaces from various positions within the testes were swabbed using cotton tipped swabs which were placed into 3 mL of phosphate buffered gelatin saline (PBGS) and tested with a pan-Pestivirus-reactive qRT-PCR.

### 2.2. Serology

A virus neutralisation test [20,22] based on a non-cytopathogenic Australian reference strain of BVDV (Trangie, subtype 1c) [23,24] was used as the primary assay for the detection of anti-Pestivirus antibodies. In some instances, an agar gel immunodiffusion assay [22] had been used to test samples prior to the introduction of the international protocol for the screening of bulls entering semen collection centres.

### 2.3. Virus and Nucleic Acid Detection

Serum from each of the bulls was tested to determine if the bull was persistently infected with BVDV using a commercial antigen capture ELISA (IDEXX BVDV Ag/Serum Plus, IDEXX Laboratories, Liebefeld-Bern, Switzerland) run according to the manufacturer’s instructions.

The detection of BVDV RNA was performed by qRT-PCR as described previously [25]. Briefly, total nucleic acid was extracted from semen samples with a magnetic particle handling system, using a volume of 25 µL of undiluted semen and 50 µL of semen diluted 1/4 in PBGS. An exogenous internal control was included in the extraction buffer for each sample. The qRT-PCR utilised pan-Pestivirus reactive primers and probe [26] run on an ABI 7500 thermocycler in standard mode for a total of 45 cycles. Cycling conditions were as specified by the Ag-Path master mix manufacturer (Ambion, Austin, TX, USA). The fluorescence threshold was set manually at 0.05 and background was automatically adjusted. qRT-PCR results were expressed as cycle threshold (Ct) values and classified as negative if no amplification was observed after 45 cycles.

Semen samples were processed [20] and virus isolation (VI) was conducted with primary bovine testis cells grown in Basal Medium Eagle (BME) with Hank’s salts containing antibiotics and supplemented with serum from BVDV antibody- and virus-free donor animals. After primary culture, samples were passaged at weekly intervals for 2 additional sub-cultures and then screened for evidence of BVDV antigen by immunoperoxidase staining [22] using a mixture of anti-Pestivirus monoclonal antibodies (P1H11, P4G11, P1D8 and 2NB2) [27]. Virus isolation was attempted on the earliest available semen samples and then periodically throughout the sampling period when BVDV RNA had been detected by qRT-PCR.

### 2.4. Immunohistochemistry (IHC)

Sections (4 µm thickness) of formalin fixed testes from Bull 2 were cut, mounted on Superfrost Plus slides (Menzel Gläser, Thermo Fisher Scientific, Waltham, MA, USA) and oven-dried for 30 min. Sections were de-waxed in xylene and rehydrated in an ethanol series. Endogenous peroxides were then blocked with a 1.8% solution of peroxide in methanol for 20 min. Slides were washed briefly in distilled water followed by 2 × 5 min washes in phosphate buffered saline (PBS) with 0.05% Tween 20 (MP Biomedicals, Santa Ana, CA, USA). Antigens were unmasked at 37 °C for 15 min in undiluted Proteinase K (Dako, Agilent, Santa Clara, CA, USA). Sections were washed twice with distilled water and once with PBS/0.05% Tween 20 before being blocked for 1 h with 0.5% bovine serum albumin (Roche, Basel, Switzerland) and 1% normal goat serum in PBS. Following a rinse in PBS/0.05% Tween 20, 150 µL of mouse MAb 15C5 (IDEXX Laboratories, Westbrooke, ME, USA) at a 1/10 dilution was added to each section and incubated at room temperature (RT; approximately 25 °C) for 1 h. The sections were washed twice in PBS/0.05% Tween 20 and incubated in EnVision™ anti-mouse serum (Dako, Agilent, Santa Clara, CA, USA) at RT for 1 h. Following three PBS/0.05% Tween 20 washes, 200 µL Dakocytomation DAB+ (Dako, Agilent, Santa Clara, CA, USA) chromogenic solution was added to each section and developed for 10 min in the dark. Slides were rinsed with distilled water to stop the colour reaction, counterstained with Mayers haematoxylin, dehydrated and mounted with dibutylphthalate polystyrene xylene (DPX) mounting medium (Sigma-Aldrich, St. Louis, MO, USA).

## 3. Results

Bulls that enter semen collection centres are shown to not be viraemic for BVDV and neutralizing antibody titres are determined. A review of records held at EMAI indicated that during the period November 2012 to October 2019, 586 bulls were tested at EMAI for entry to artificial breeding centres. The majority of bulls in this group were *Bos taurus* breeds (95.9%), with *Bos taurus* × *Bos indicus* breeds (2.5%) and *Bos indicus* breeds (1.6%) making up the remainder. Neutralizing antibodies to BVDV were detected in 295 (50.3%) of these bulls at the time of screening. Semen samples from all bulls that had virus neutralizing antibodies to BVDV were tested by qRT-PCR.

Six bulls were identified to have PTIs because BVDV RNA was detected in semen more than 60 days after the first detection of infection. The 60-day limit provided sufficient time to elapse to exclude the possibility of residual RNA or virus from a recent acute infection being detected. All of these bulls gave negative results in the antigen ELISA, confirming that none was persistently infected. All bulls had high neutralizing antibody titres during the observation period. Live attenuated Pestivirus vaccines are not used in Australia and none of the bulls in this study had been vaccinated. Full details for the six infected bulls are described in Appendix A Table A1, Table A2, Table A3, Table A4, Table A5 and Table A6

Both beef (five) and dairy (one) bulls were involved, with ages at the time of first detection of virus infection ranging from 10 to 21 months (Table 1). BVDV RNA was detected in the semen of these bulls for periods ranging from 3 to 73 months after the latest date at which the bulls could have been infected. Due to the very low levels of RNA detected on many occasions, virus isolation was only attempted on a limited proportion of samples. BVDV was successfully isolated from 12.5% (4/32) of samples subjected to virus isolation.

The data for Bull 2 (Table A2) provides an interesting insight into PTIs. BVDV was retrospectively detected in the semen of this bull over a period of more than five years. The first detection of BVDV RNA was in 2017 during the routine screening protocol for seropositive bulls. A review of the testing history for this bull indicated that it had been infected with BVDV prior to late January 2012. Fifteen batches of semen were available over a six-month period between April and October 2012. Interestingly, when this semen was tested in 2018, BVDV RNA was only detected in the last of these 15 samples (26 October 2012). After the detection of BVDV RNA in May 2017, 10 consecutive positive semen samples were identified in 2018, followed by two negative semen samples prior to this bull being sent to an abattoir. No semen was available for testing between October 2012 and May 2017.

**Figure 1:**
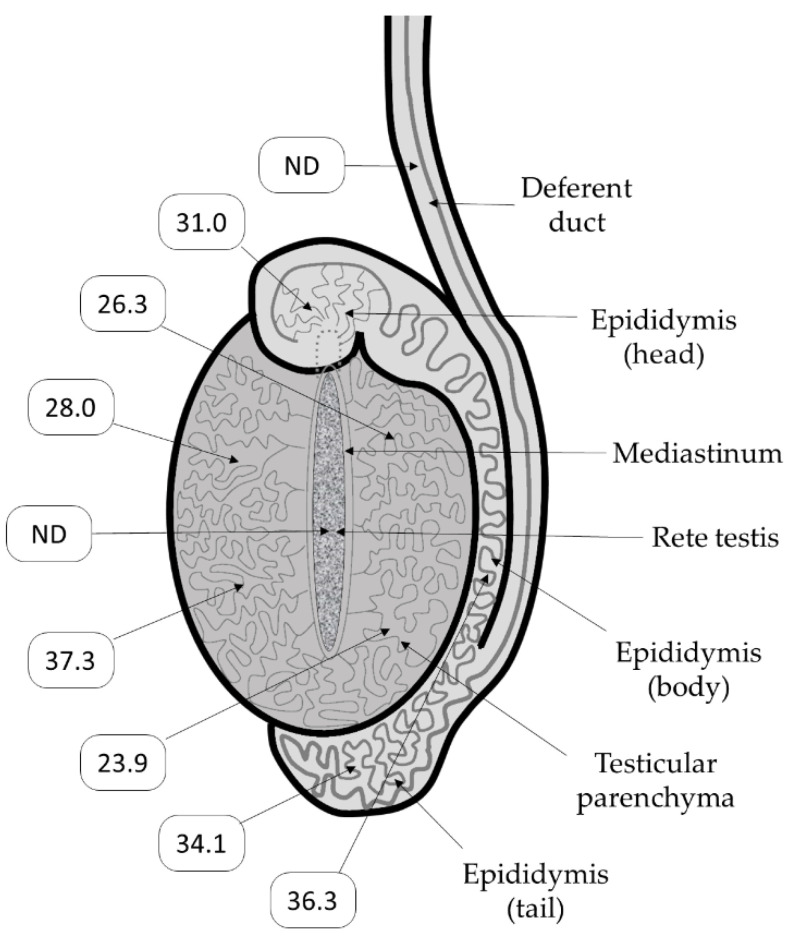
Schematic view of the affected testis of Bull 2. The numbers indicate the Ct values of swabs taken from the sites indicated by arrow heads. ND = BVDV RNA was not detected.

Each of these bulls with a presumed PTI was clinically normal and without any physical abnormalities, with the exception of Bull 2. Soon after arriving at the artificial breeding centre, this bull had scrotal measurements recorded on two occasions, with a circumference of 31 cm on 20 February 2012 and 34 cm on 16 April 2012. These measurements were considered normal for a bull of his breed and age [28]. Six years later, Bull 2 was sent an abattoir in June 2018. The testes from this bull were forwarded to EMAI where they were measured, weighed and dissected. One testis was 20 cm long and weighed 720 g. The other was 16 cm long and weighed 431 g. Swabs were taken from freshly cut surfaces at various locations within each testis and qRT-PCR performed. The 16 swabs taken from the larger of the testes gave negative qRT-PCR results, while positive results were obtained from seven of the nine swabs taken from the smaller one. The highest levels of BVDV RNA (as indicated by the lowest Ct values) were found in swabs taken from the parenchyma of the testis (Figure 1). However, this result was not uniform across the testicle with one of the four parenchymal swabs yielding a very high Ct value (37.3). Lower levels of BVDV RNA were detected along the epididymis and no viral RNA was detected in the deferent duct or the rete testis (Figure 1). BVDV was isolated from the testicular parenchyma.

**Figure 2:**
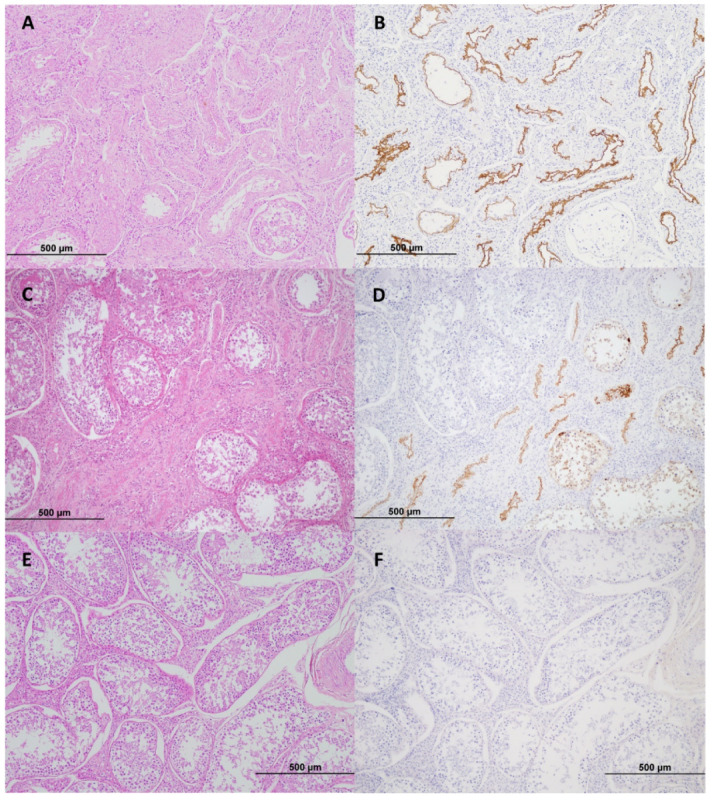
(**A**) Locally extensive areas of seminiferous tubular atrophy and fibrosis in a BVDV-infected bovine testicle. Many tubules are collapsed and shrunken with total loss of Sertoli cells, with a few tubules retaining some Sertoli cells but no germinal cells. The lower right quadrant contains a tubule with germinal cells. Haematoxylin and eosin (H&E) stain, 10×. (**B**) Non-serial section of A. Collapsed tubules have circumferential profiles of intense BVDV staining at the luminal surface. BVDV immunohistochemistry (IHC), 10×. (**C**) Collapsed seminiferous tubules intermingle with less-affected or relatively histologically normal tubules (lower right and upper left, respectively) in a BVDV-infected testicle. H&E stain, 10×. (**D**) Serial section of C; Collapsed tubules have intense BVDV staining while adjacent less-affected tubules (lower right) have BVDV staining within still-existing germinal cell stages and Sertoli cells. Histologically normal tubules (upper left) are BVDV antigen negative. BVDV IHC, 10×. (**E**) Histologically normal testicular tissue in the BVDV PCR negative contralateral testicle from Bull 2. H&E stain, 10×. (**F**) Serial section of E. The contralateral testicle is BVDV antigen negative. BVDV IHC, 10×.

**Figure 3:**
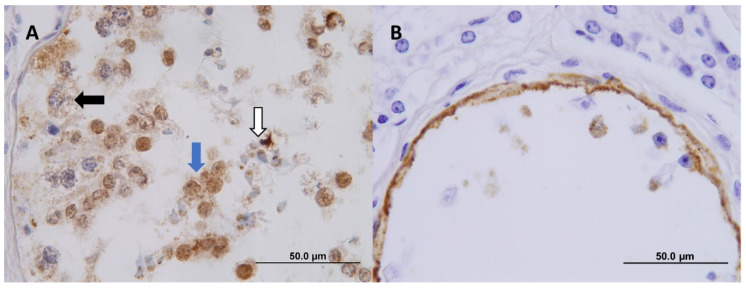
(**A**) A seminiferous tubule containing still-existing germinal cell stages in a BVDV-infected bovine testicle. BVDV staining is present in cells morphologically consistent with primary spermatocytes (black arrow), round spermatids (blue arrow) and elongated spermatids (white arrow). BVDV IHC, 100× (oil). (**B**) An atrophic seminiferous tubule in a BVDV-infected bovine testicle. The tubular luminal surface has intense BVDV staining which appears to occasionally extend into the superficial tubular mesenchyme and within degenerate free intraluminal cells. BVDV IHC, 100× (oil).

When examined microscopically, the abnormal testis from Bull 2 showed multifocal but locally extensive areas of seminiferous tubular atrophy (Figure 2A,C) characterised by reduced or absent germinal cell stages and aspermia. Affected tubules had either a reduced lumen diameter lined by a variably intact Sertoli cell layer or else were collapsed with complete or near total loss of Sertoli cells. Tubular atrophy was accompanied by moderate to marked tubular hyalinization and fibrosis and an increased prominence of Leydig cells. Immunohistochemical staining for BVDV antigen in these areas showed intense circumferential staining of the tubular luminal surface (Figure 2B and Figure 3B). Within these tubules, there was also occasional staining of the subjacent thickened tubular wall mesenchyme and rarely within the cytoplasm of free intraluminal degenerative cells (Figure 3B). Adjacent to some areas of collapsed tubules were small clusters of less-affected tubules (Figure 2C) with low or no BVDV staining at the tubular luminal surface but containing numerous identifiable germinal cell stages which contained BVDV staining, including primary spermatocytes, round spermatids and elongate spermatids (Figure 3A). There was no evidence of inflammation associated with the affected tubules. Areas predominated by tubular lesions containing BVDV staining often intermingled with nearby histologically relatively normal tubules in which BVDV staining was not detected (Figure 2D). Representative sections of the epididymis were examined, found to be histologically unremarkable and BVDV IHC negative. The contralateral testicular tissue was histologically within normal limits (Figure 2E) and BVDV IHC negative (Figure 2F).

## 4. Discussion

International semen collection protocols and the associated health certification of donor bulls [21] are designed to ensure that bovine semen is free of contamination with BVDV. In addition to the exclusion of PI animals, measures are aimed at the exclusion of both transiently infected bulls and those with PTI. It was the adoption of these measures that resulted in the detection of the PTI bulls that are the subject of this report, and the detection of contaminated batches following retrospective testing. This study adds to the collective knowledge of prolonged or persistent testicular infection (PTI) in bulls through the identification and detailed investigation of six bulls. The longitudinal sampling, histopathology and IHC results have increased our understanding of the pathology and pathogenesis of PTI.

Studies of transiently infected bulls in the past have relied on virus isolation for the detection of BVDV in the semen. The use of qRT-PCR to detect BVDV in semen is recommended by the OIE [20], yet there is a lack of published literature on investigations of either transiently infected bulls or those with a PTI using qRT-PCR. It is not clear precisely how long BVDV RNA may be detected in semen following a transient infection. The possibility that some of these bulls were undergoing a transient infection when BVDV RNA was detected in the semen has to be considered due to the higher sensitivity of qRT-PCR, which may detect virus for a much longer time than virus isolation [26]. The date when Bull 5 was first infected can be estimated with the greatest precision due to the rising neutralizing antibody titre on the first two serum samples. Notably, the first semen sample to be tested gave a positive result and this sample was collected at least two months after the likely time of infection. Additionally, positive qRT-PCR results were obtained for semen samples for another two months. Therefore, it would seem unlikely that this RNA detection was the product of a transient infection. Similarly, with the exception of Bull 2, the other bulls were observed to shed viral RNA in semen for periods between three and eight months. Bull 2 shed virus for more than five years. Each of these bulls was likely to have experienced PTIs because the length of these shedding periods is in marked contrast to the 10 to 14 day periods reported for transiently infected bulls [9,10].

In 1998, Voges et al. described a post-pubertal bull that was shown to shed BVDV in semen over an 11-month period. This bull had a consistently high antibody titre against BVDV and yet was not viraemic and virus could not be detected in any organ apart from the testes. This bull was described as being in good health until his slaughter at 22 months of age. His growth rate and testicular development were unremarkable. Two other cases of PTI in bulls have been described in 2005 and 2014. [17,19]. In the latter report, a post-pubertal bull was also shown to be not PI. Infectious virus and viral RNA were detected in this bull up to 42 months of age. Virus was not detected for the last six months of this bull’s life. IHC on this bull’s testes at 48 months of age failed to detect viral antigen.

Voges et al. [18] proposed that for PTI to occur, BVDV infection should occur shortly before puberty, however, the pathogenesis of prolonged or persistent testicular infection is not clear. In cattle, puberty is evaluated on the basis of testicular growth and quantitative measures of sperm production [29]. Published estimates for the average age at puberty in bulls put this at approximately 10.5 months, ranging from 9.4 to 12.1 months [30,31,32]. It is of interest to note that five of the six bulls in our study were in the range of 17–21 months old when virus infection was first detected. The age at which three bulls seroconverted could be determined relatively accurately (Bulls 4, 5 and 6). These bulls were between 18 and 20 months of age when infection was first detected. Bull 2 was less than 10 months of age when he was exposed to BVDV. The age at which the other two bulls seroconverted to BVDV could not be accurately determined but their virus detection results indicate an upper age limit (18–21 months) at which infection may have occurred and is similar to the age at which the other three bulls became infected. The results of the three bulls whose age at infection could be estimated with some reliability challenges the notion that infection of PTI bulls only occurs at or around puberty.

The localization of BVDV in the reproductive tract of Bull 2 was different to what had been observed for transiently infected bulls. BVDV RNA was only detected in the smaller of the testes. Relatively high levels of RNA and antigen were detected in the parenchyma of this infected testis and BVDV was also isolated in cell culture. This is in contrast to what had been observed for transiently infected bulls, where virus was not isolated from the testicular parenchyma. In this bull, trace levels of BVDV RNA were also detected in the epididymis but, unlike observations of transiently infected bulls, no antigen was detected. Unfortunately, the accessory glands of Bull 2 were not examined, preventing further comparisons with the observations made in transiently infected bulls where virus was only isolated from the accessory glands.

The pathology and IHC staining of Bull 2 clearly demonstrated that the persistence of the BVDV infection was localized within the immune-privileged seminiferous tubule compartment. The bovine testis consists of two compartments, the peritubular compartment (Leydig cells and testicular macrophages) and the seminiferous tubule compartment (germ cells protected by Sertoli cells). The Sertoli cells span the basal to adluminal sections of the tubule and tight junctions between these cells form the blood–testes barrier [33]. This barrier protects developing germ cells from stimulating the adaptive immune system while allowing nutritional and structural support [34]. The peritubular compartment is exposed to the full immune response of the animal, while the seminiferous tubule compartment is in an immune-privileged site [35].

The pattern of pathology detected in this testis shows a continuum of tubular degenerative change correlating closely with a progression of BVDV staining, suggesting that the infection in this case was confined to seminiferous tubules and chronicity resulted in the eventual destruction of the normal tubule structure. It is also noteworthy that the concentration of BVDV RNA was not uniform across the testis. Within one area of the testicular parenchyma, the qRT-PCR gave a Ct value of 37.3. This result indicates that the level of BVDV RNA present was very close to the limit of detection for this assay. Yet, in the other areas of the testicular parenchyma tested, the Ct values ranged from 23.9 to 28.0. These values are typically seen in the semen of PI cattle. We speculate that the infection in PTI remains largely hidden from the immune system and manages to smoulder within the seminiferous tubules for a variable amount of time. The case of the bull from which the testes were collected at the abattoir supports this notion as there was a lack of interstitial or tubular inflammatory cellular infiltrates in response to the widespread presence of BVDV within the tubules, even in areas where BVDV tubule staining appears to extend superficially into the subjacent thickened peritubular mesenchyme of intensely stained tubules This latter observation may be a by-product of excessive antigen accumulation in these tubules, or perhaps the peritubular fibrosis assisted to shelter the infection from the peritubular compartment immune response. We speculate that the destruction of the tubules contributed to the small overall size of this testis. It was not possible to determine if the uninfected testis was ever infected, though no histological signs of past infection were detected. BVDV RNA was only detected in one of the early semen collections in 2012. It is not clear if BVDV RNA levels were too low to detect in these collections, or if there was deterioration in the RNA over the six years between when the RNA was shed and when it was tested by qRT-PCR. Additionally, the final two semen collections for this bull gave negative results in the qRT-PCR. Nevertheless, the ability to isolate virus from the testis of this bull 73 months after the initial infection demonstrates the capacity of the infection to persist in the testis for long periods.

The time course over which a PTI may be detected appears to range from a few months to many years. Eventually infection may be cleared, as appeared to occur in Bull 5 and as described by Newcomer et al., [17], or infection may remain persistent as demonstrated by Bull 2. Distinctions between persistent testicular infections and prolonged testicular infections have been proposed based on the ability to infect susceptible animals [17]. This distinction has not been applied to this series of bulls. BVDV was isolated from three of the six bulls (Bulls 2,4 and 6). Moreover, BVDV was isolated from the semen of Bull 2 on two occasions but was not isolated on eight. It is probable that differences between the ability to detect BVDV by qRT-PCR and by VI reflect shedding of the virus in semen, which in turn may vary between semen batches and progress of the infection.

Virus isolation has been considered the gold standard for BVDV detection when screening semen for the presence of BVDV-1. However, the results of the current investigations demonstrate the benefits of using qRT-PCR, which offers both higher analytical sensitivity and speed for the detection of BVDV infection. It is important that qRT-PCR assays employ broadly reactive pan-Pestivirus primers and probes to ensure that the potential genetic variability of all strains of Pestiviruses are reliably detected [20]. Many of the Ct values recorded for these positive bull semen samples were relatively high. The work of Hoffman et al. demonstrated that, for qRT-PCR assays, Ct values greater than 38 represent less than 10 genome copies per 5 µL of sample [26]. However, the volume of sample should not be overlooked because detection of 10 copies in a qRT-PCR assay represents approximately 1000 copies of RNA in a single 500 µL straw of semen. Parallel qRT-PCR and virus isolation experiments demonstrated that qRT-PCR had 100 to 1000 times greater sensitivity in contrast to the limit of detection for virus isolation methods. While qRT-PCR does not provide an indication of whether the sample contains infectious virus, a positive result in this assay does identify a batch as presenting a risk that infectious virus may be present. This risk is exemplified by experimental work [14] that demonstrated that, despite BVDV being detected by PCR, virus isolation attempts in cell culture with the semen of PTI bulls were unsuccessful. This same semen was capable of producing a systemic infection when injected into calves. Therefore, as recommended by the OIE [20], a reliable qRT-PCR should be the preferred diagnostic assay for BVDV detection for the screening of semen. Diagnostic laboratories are encouraged to undertake regular evaluation of any qRT-PCR assays that are being used to detect BVDV. This evaluation should involve comparison of published assays considering both the nucleic acid extraction and qRT-PCR components, using suitable samples from the country of origin, assessing the limit of detection and participating in proficiency testing.

With an increasing number of countries progressing towards freedom from BVDV, consideration may need to be given to screening of batches of stored semen. Most collections of semen from bulls that experience a PTI have very low levels of infectious virus and perhaps often at levels that would be lower than an infective dose following insemination. Nevertheless, this semen does present a biosecurity risk. Indeed, it had been shown that use of semen from the bull with the first recognized PTI was able to establish a systemic infection in an inseminated heifer [36], thereby confirming this risk. Contaminated semen can be used many years after collection and is often transported long distances internationally. These factors, combined with the potential for exposure of a large number of breeding females to a single batch of semen, increase the potential for the introduction of BVDV into free herds and countries, or potentially the introduction of other species of Pestivirus (such as BVDV-2 and Hobi-like Pestiviruses) into countries where these viruses are absent and where appropriately protective vaccines are not used.

In conclusion, although prolonged or persistent testicular infections are a relatively rare event, the findings of this study emphasize the recommendations [20] for all batches of semen from seropositive bulls to be screened to exclude the possibility of BVDV. Further, it is recommended that this screening is more effectively carried out by qRT-PCR rather than by virus isolation methods.

## Figures and Tables

**Table 1:** Summary of details for bulls studied.

Bull Number	Breed	Estimated Age at First Detection of Infection (Months) ^a^	Estimated Duration of Shedding (Months) ^b^	qRT-PCR Results in Semen (Ct)	Virus Isolation (Pos/Total Tested)
1	Main Anjou	21	3	32.8–35.8	0/6
2	Holstein	10	73	29.7–38.4	2/10
3	Angus	18	3	29.1–32.6	0/5
4	Angus	19	4	30.1–32.0	1/3
5	Angus	20	6	32.6–34.8	0/6
6	Wagyu	21	8	27.6–38.0	1/2 ^c^

^a^ Detection of seroconversion or detection of bovine viral diarrhoea virus (BVDV) RNA in semen, whichever occurred earlier. ^b^ Time between detection of infection and last detection of BVDV RNA in semen. ^c^ Virus isolation successful in the early phase of infection.

## Appendix A

**Table A1:** Serological and virological test results for Bull 1.

Sample Date	Sample Type	Age (Months)	qRT-PCR (Ct)	Virus Isolation	Antigen ELISA	VNT (Titre)
28/08/2012	Serum	21			Negative	512
22/10/2012	Serum	23		Negative		≥512
12/11/2012	Extended semen	23	Positive (35.4)	Negative		
23/11/2012	Extended semen	24	Positive (35.4)	Negative		
26/11/2012	Extended semen	24	Positive (35.8)	Negative		
28/11/2012	Extended semen	24	Positive (32.8)	Negative		
30/11/2012	Extended semen	24	Positive (35.5)	Negative		
6/12/2012	Serum	24				≥512

Breed: Maine-Anjou. Date of birth: December 2010. Time of infection: prior to 28 August 2012. VNT—virus neutralisation titre.

**Table A2:** Serological and virological test results for Bull 2.

Sample Date	Sample Type	Age (Months)	qRT-PCR (Ct)	Virus Isolation	Antigen ELISA	AGID	VNT (Titre)
23/01/2012	Serum	10			Negative	3	
13/03/2012	Serum	12			Negative	2	
17/04/2012	Extended semen	13	Negative ^a^	Negative			
17/04/2012	Extended semen	15	Negative ^a^				
19/06/2012	Extended semen	15	Negative ^a^				
29/06/2012	Extended semen	16	Negative ^a^				
20/07/2012	Extended semen	17	Negative ^a^				
8/08/2012	Extended semen	17	Negative ^a^				
20/08/2012	Extended semen	17	Negative ^a^				
24/08/2012	Extended semen	17	Negative ^a^				
31/08/2012	Extended semen	18	Negative ^a^				
4/09/2012	Extended semen	18	Negative ^a^				
7/09/2012	Extended semen	18	Negative ^a^				
11/09/2012	Extended semen	18	Negative ^a^				
21/09/2012	Extended semen	18	Negative ^a^				
28/09/2012	Extended semen	19	Negative ^a^				
26/10/2012	Extended semen	20	Positive (36.5) ^a^				
13/11/2012	Serum	26			Negative	>3	
7/05/2013	Serum	38			Negative	3	
5/05/2014	Serum	50			Negative	3	
4/05/2015	Serum	62			Negative	3	
2/05/2016	Serum	74			Negative		≥512
1/05/2017	Extended semen	82	Positive (29.7)	Positive			
18/01/2018	Extended semen	82	Positive (32.6)	Negative			
23/01/2018	Extended semen	82	Positive (32.4)	Negative			
25/01/2018	Extended semen	83	Positive (30.9)	Positive			
1/02/2018	Extended semen	83	Positive (35.9)	Negative			
6/02/2018	Extended semen	83	Positive (32.5)	Negative			
8/02/2018	Serum	83			Negative		
12/02/2018	Extended semen	83	Positive (33.3)	Negative			
13/02/2018	Extended semen	83	Positive (34.4)	Negative			
15/02/2018	Extended semen	83	Positive (31.58)	Negative			
16/02/2018	Extended semen	83	Positive (38.46)				
20/02/2018	Extended semen	83	Positive (36.82)				
22/02/2018	Extended semen	83	Negative				
27/02/2018	Extended semen	84	Negative				

Breed: Holstein. Date of birth: March 2011. Time of infection: prior to 23 January 2012. ^a^ Tested in 2018. AGID—agar gel immunodiffusion assay

**Table A3:** Serological and virological test results for Bull 3.

Sample Date	Sample Type	Age (Months)	qRT-PCR (Ct)	Virus Isolation	Antigen ELISA	VNT (Titre)
20/02/2013	Serum	15			Negative	
22/05/2013	Extended semen	18	Positive (29.2)			
24/05/2013	Extended semen	18	Positive (29.7)	Negative		
28/05/2013	Extended semen	18	Positive (29.3)	Negative		
30/05/2013	Extended semen	18	Positive (29.1)			
3/06/2013	Extended semen	18	Positive (32.6)			
7/06/2013	Extended semen	18	Positive (30.0)			
10/06/2013	Serum	18		Negative		≥512
12/06/2013	Extended semen	18	Positive (31.0)			
14/06/2013	Extended semen	19	Positive (30.5)			
18/06/2013	Extended semen	19	Positive (29.5)			
20/06/2013	Extended semen	19	Positive (30.0)			
24/06/2013	Extended semen	19	Positive (30.4)			
26/06/2013	Extended semen	19	Positive (30.5)			
3/07/2013	Extended semen	19	Positive (30.5)			
8/07/2013	Extended semen	19	Positive (32.0)			
12/07/2013	Extended semen	19	Positive (31.0)			
12/07/2013	Extended semen	19	Positive (30.5)	Negative		
16/07/2013	Extended semen	20	Positive (30.4)			
18/07/2013	Extended semen	20	Positive (31.4)			
23/07/2013	Extended semen	20	Positive (32.2)			
15/08/2013	Extended semen	21	Positive (30.7)	Negative		

Breed: Angus. Date of birth: December 2011. Time of infection: prior to 22 May 2013.

**Table A4:** Serological and virological test results for Bull 4.

Sample Date	Sample Type	Age (Months)	qRT-PCR (Ct)	Virus Isolation	Antigen ELISA	AGID	VNT (Titre)
3/09/2013	Serum	18			Negative	Negative	
8/10/2013	Serum	19			Negative	>3	
22/10/2013	Extended semen	20	Positive (31.7)	Positive			
24/10/2013	Extended semen	20	Positive (32.0)	Negative			
16/12/2013	Seminal fluid	22	Positive (30.2)	Negative			
16/02/2014 ^a^	Extended semen	24	Positive (30.1)				
16/02/2014 ^a^	Serum	24					≥512

Breed: Angus. Date of birth: March 2012. Time of infection: between late August and September 2013. ^a^ Slaughtered immediately after this collection.

**Table A5:** Serological and virological test results for Bull 5.

Sample Date	Sample Type	Age (Months)	qRT-PCR (Ct)	Virus Isolation	Antigen ELISA	VNT (Titre)
31/03/2014	Serum	20			Negative	4
12/05/2014	Serum	21		Negative		256
15/05/2014	Extended semen	21	Positive (32.6)			
24/05/2014	Neat semen	22	Positive (34.2)	Negative		
11/06/2014	Extended semen	22	Positive (34.8)	Negative		
2/07/2014	Neat semen	23	Positive (34.1)	Negative		
17/07/2014	Extended semen	23	Positive (33.9)	Negative		
20/02/2015	Serum	31			Negative	≥512
8/04/2015	Extended semen	32	Negative			
20/04/2015	Serum	32		Negative		≥512
22/04/2015	Extended semen	33	Negative			
27/04/2015	Extended semen	33	Negative			
14/05/2015	Extended semen	33	Negative			
19/05/2015	Extended semen	33	Negative			
21/05/2015	Extended semen	34	Negative			
25/05/2015	Extended semen	34	Negative			
28/05/2015	Extended semen	34	Negative			
2/06/2015	Extended semen	34	Negative			
4/06/2015	Extended semen	34	Negative			
8/06/2015	Extended semen	34	Negative			
12/06/2015	Extended semen	34	Negative			
16/06/2015	Extended semen	34	Negative			
28/06/2015	Extended semen	35	Negative			
18/07/2015	Extended semen	35	Negative			
17/08/2015	Extended semen	36	Negative			
21/08/2015	Extended semen	37	Negative			
24/08/2015	Extended semen	37	Negative			
31/08/2015	Extended semen	38	Negative			
6/02/2017	Serum	54			Negative	512
3/04/2017	Serum	56				128
1/05/2017	Extended semen	57	Negative			
4/05/2017	Extended semen	57	Negative			

Breed: Angus. Date of birth: August 2012. Time of infection: probably infected mid-March 2014.

**Table A6:** Serological and virological test results for Bull 6.

Sample Date	Sample Type	Age (Months)	qRT-PCR (Ct)	Virus Isolation	Antigen ELISA	VNT
14/12/2018	Extended semen	15	Negative			
6/04/2019 ^a^	Serum	19			Negative	
21/05/2019	Extended semen	20	Positive (27.6)	Positive		
1/09/2019	Serum	24			Negative	>512
24/09/2019	Serum	24				>512
17/10/2019	Extended semen	25	Positive (38.0)	Negative		
22/11/2019	Extended semen	26	Positive (36.4)			
8/01/2020	Extended semen	28	Positive (36.2)			
23/01/2020	Extended semen	28	Negative			

Breed: Wagyu. Date of birth: September 2017. Time of infection: estimated infected between mid-April and mid-May 2019. ^a^ A BVDV antibody ELISA was performed on serum taken on 6 April 2019 with negative results (data not shown).
